# The emerging therapeutic role of some pharmacological antidotes in management of COVID-19

**DOI:** 10.1186/s43168-021-00105-7

**Published:** 2022-01-20

**Authors:** Doaa M. El Shehaby, Marwa Kh. Mohammed, Noha Esmael Ebrahem, Mariam M. Abd El-Azim, Islam G. Sayed, Sarah A. Eweda

**Affiliations:** 1 Forensic Medicine and Clinical Toxicology Department, Faculty of Medicine, Assiut University, Asyut, Egypt; 2 Chest Department, Faculty of Medicine, Aswan University, Aswan, Egypt; 3 Forensic Medicine and Clinical Toxicology Department, Faculty of Medicine, Ain Shams University, Cairo, Egypt

**Keywords:** COVID-19, NAC, HBO, Deferoxamine, LDN

## Abstract

**Background:**

A novel RNA coronavirus was identified in January 2020 as the cause of a pneumonia epidemic affecting the city of Wuhan; it rapidly spread across China.

**Aim of the review:**

The aim is to discuss the potential efficacy of some pharmacologically known pharmacological antidotes (N-acetylcysteine; hyperbaric oxygen; deferoxamine; low-dose naloxone) for the management of COVID-19-associated symptoms and complications.

**Method:**

An extensive search was accomplished in Medline, Embase, Scopus, Web of Science, and Central databases until the end of April, 2021. Four independent researchers completed the screening, and finally, the associated studies were involved.

**Conclusion:**

The current proof hinders the experts for suggesting the proper pharmacological lines of treatment of COVID-19. Organizations, for example, WHO, should pursue more practical actions and design well-planned clinical trials so that their results may be used in the treatment of future outbreaks.

## Introduction

Early in January 2020, a novel RNA coronavirus was identified as the cause of a pneumonia epidemic affecting Wuhan, spreading rapidly across China, then across the world, with the quickly increasing every day number of established new cases [1–3]. Up to 28 March 2020, the death of 26,495 individuals worldwide and infection of more than 570,000 were caused by COVID-19 [4].

The World Health Organization (WHO) named coronavirus disease 2019 as COVID-19. It has been declared as a pandemic because of its widespread infectivity and highly contagious rate. Respiratory and enteric infections are the typical presentation of the human coronaviruses [5, 6]. The main presentation of infection with COVID-19 is the flu-like symptoms such as fever, cough, and asthenia, resembling other coronaviruses [7, 8]. Severe lung injury has been pronounced at all ages, which can precipitate acute respiratory failure with high fatality rates. The virus is more probable to cause severe manifestations in some high-risk individuals, such as the elderly or those affected by multiple previous morbidities, these in the form of acute respiratory distress syndrome, interstitial pneumonia, and subsequent multi-organ failure [9].

COVID-19 is mainly a disease with respiratory manifestations, but it is important for clinicians to be aware of the increasing reports of thrombotic and cardiovascular complications which are recognized during infection with COVID-19. The proinflammatory immune response with high levels of inflammatory processes which is present in most infected cases is associated with hypercoagulable state [10].

Management of the infected cases is mainly symptomatic and supportive [11–14]. Most available therapeutic options for managing COVID-19 are based on the experiences regarding treatment of SARS-CoV and MERS-CoV. The WHO guidelines of treatment of infected cases are supportive care including oxygen therapy in addition to fluid therapy and antibiotics for treating secondary infections, with social isolation of the confirmed and suspected patients for COVID-19 [15].

The purpose of the present mini-review is to discuss the potential efficiency of some pharmacologically known antidotes in alleviating the clinical manifestations of COVID-19 contagion with a brief discussion of their mechanisms to counteract the symptoms of COVID-19 infection.

## Main text

### Methodology

Two groups of investigators were working independently for the study selection. A search was performed using the following databases: Science Direct, PubMed, Scopus, Medline, Google Scholar, and Web of Science. Any discrepancies were resolved through consensus. All articles which were supposed potentially authorized were retrieved for full-text reviews. We limited our search results to publications in English and excluded abstracts from conferences and observations. The keyword “coronavirus” was paired with coronavirus and/or COVID-19 was paired with one or more of “ARDS,” “N acetyl cysteine,” “hyperbaric oxygen,” “deferoxamine,” “low dose naloxone,” and “methylene blue” to obtain published articles up till October 2020. No language restraint was imposed.

## Discussion

The COVID-19 pandemic is caused by the coronavirus 2 (SARS-CoV-2) that belongs to the Coronaviridae family. This family has been accountable for two viral epidemics recently; the first one was during 2003 due to severe acute respiratory syndrome coronavirus (SARS) [16]. The second outbreak was during 2012 due to the spread of coronavirus (MERS-CoV) [17].

Recently, severe acute respiratory syndrome (SARS-CoV-2), a novel strain of fatal coronavirus, struck China mainly in Wuhan. It is a beta type of coronaviruses proposed with the name (family: Coronaviridae). This novel strain of coronavirus managed to spread within a very short period of time over a wide geographic location. On February 28, 2020, the total number of confirmed nCoV-19 infections worldwide was 83,652, and the number of deaths is more than 3000 [18].

Coronaviruses (CoV) are enveloped viruses that contain non-segmented, positive-stranded genomic RNA [19]. These viruses are characterized by being pleomorphic particles, sized from 80 up to 120 nm, and their replication cycle entirely occurs in the cytoplasm [20]. An innovative coronavirus 2019 (nCoV-19) has been recently identified in humans that was responsible for thousands of deaths during the period from January to March 2020 [21]. Furthermore, CoVs were established to be the causal of the Middle East respiratory syndrome (MERS-CoV) and SARS-CoV-2. Serologically, there are three strains of this virus that have been reported up to date. Two strains which are HCoV-229E and HCoV-OC43 have been identified in 1960 causing the well-controlled common cold symptoms. The third coronavirus that is life-threatening and may lead to lethal pneumonia is called SARS-CoV [22].

COVID-19 virus transmission can occur primarily direct through contact with the diseased cases or indirect through contact with objects used by the infected people [5, 6, 23]. Respiratory contagions can be occur through droplets of diverse sizes (droplet particles; sized > 5–10 μm and droplet nuclei; sized < 5 μm) [24]. Studies on cultured COVID-19 virus from a stool specimen revealed its feco−oral transmission [25, 26].

The sequence of the virus life within the host consists of 5 stages: attachment, penetration, biosynthesis, then maturation and lastly release. At first (attachment) is the binding of the viruses to the host receptors, entering host cells through membrane fusion (penetration) or endocytosis. Immediately when the viral contents were released inside the host cells, the viral RNA enters the nucleus for replication. Viral mRNA is used to make viral proteins (biosynthesis). Finally, the novel viral particles are designed (maturation) and released [27].

### Therapeutic management of COVID-19

Presently, there is no evidence from randomized clinical trials (RCTs) that any potential therapy improves outcomes in patients with either suspected or confirmed COVID-19. There are no clinical trial data supporting any prophylactic treatment. More than 3 hundred vigorous clinical treatment trials are in progress [28].

The treatment protocols for severe cases of SARS include supportive care with mechanical ventilation and ICU admission [29], depending on the hypothesis of cytokine deregulation, and treatment guidelines including the administration of steroids, aiming to modulate the exacerbated cytokine response [30].

Recent guidelines establish the usage of some antibacterial drugs for prevention of secondary bacterial contagions and steroids for modulation of cytokine deregulation in addition to ribavirin which is a nucleoside analog with a broad antiviral activity [25, 26]. Several challenges were made to study in vitro susceptibility to various complexes with potential anti-SARS activity.

#### Hyperbaric oxygen therapy

Hyperbaric oxygen therapy (HBOT) is the primary antidotal therapy for acute carbon monoxide toxicity. It is a sort of management considered to increase the oxygen level in the blood delivered to the tissues. It is defined as breathing of 100% oxygen at elevated atmospheric pressure than the sea-level. The pressure applied usually is 2 to 3 times the atmospheric pressure at sea level [31]. It can be delivered either in a mono-place or a multi-place chamber. Mono-place chambers are condensed with pure oxygen accepting single case at one time, while multi-place chambers are pressurized with air accommodating multiple cases that can breathe throughout a tight-fitting face or endotracheal tube as clinically indicated. Management generally lasts for up to 8 h, depending on the indication, and may be accomplished 1 to 3 times on a daily basis [32].

Presently, there are numerous FDA accepted circumstances for the practice of HBOT in clinical toxicology as carbon monoxide poisoning, as well as gas and air embolism, clostridial myositis, crush injury, compartment syndrome, decompression sickness, diabetic foot, and chronic intractable osteomyelitis [33].

Therapeutic mechanisms of action of HBOT are established on Henry’s Law, which declares that the concentration of an interfacing gas (oxygen in the alveoli of the lungs) in a liquid (pulmonary blood) is comparative to the interfacing gas pressure. The final oxygen uptake and binding to the hemoglobin in red blood cells of the pulmonary capillary are reliant on the diffusion of dissolved oxygen across alveolar-capillary membrane to blood flow [34].

Hyperbaric oxygen therapy produces the rise of both the hydrostatic pressure and the partial pressure of oxygen during which the arterial oxygen tension classically exceeds 1500 mm Hg, consequently rising the dissolved oxygen content of plasma over the required to meet the cellular resting requirements without any involvement from the hemoglobin binding oxygen [32].

##### Rationale of hyperbaric oxygen therapy in management of COVID-19

Progressive hypoxemia is a characteristic sign in the clinical course of severe COVID-19 pneumonia. The latest data suggest that interstitial and alveolar inflammation amid the thickened alveolar-capillary membrane was the major pathological alteration in COVID-19 pneumonia [35, 36]. Moreover, systemic metabolic rate was found to be continuously rising owing to the systemic inflammation, so the amount of oxygen transported by hemoglobin cannot meet the body’s metabolic needs. The body is in a “chronic” hypoxic state of systemic tissues; therefore, in some patients, the extracorporeal membrane oxygenation may still be insufficient to correct hypoxia in deep tissues and vital organs [37, 38].

Several studies suggest certain proteins of the novel virus could attack the 1-beta chain of hemoglobin, consequently dissociating iron from porphyrin resulting in destruction of the hemoglobin as a cause of hypoxemia in COVID-19. This attack would result in a drop in the hemoglobin available to carry oxygen and also shift the oxygen dissociation curve to the left hence producing a picture comparable to carbon monoxide poisoning [39].

The application of HBOT to pneumonia cases due to COVID-19 is of great effectiveness through the various following mechanisms:

1. It enhances multiple stages of oxygen diffusion by increasing the dissolved oxygen in the alveolar and inflammatory barriers with subsequent increasing the amount of oxygen dissolved in blood plasma, so raising the oxygen saturation of hemoglobin in red blood cells. Thus, it delivers satisfactory blood oxygen levels virtually in comprehensive absence of lung-blood exchange [40]. Moreover, it solves the imbalance between oxygen prerequisite and oxygen available through providing the body with an intermission of adequate aerobic metabolism for the deep hypoxic tissues and important organs playing a good role in supporting treatment [41].
2. It has a definite immunosuppressive effect; it can reduce the intensity of the inflammatory response to stimulus-induced pro-inflammatory cytokine construction. It encourages cytokine downregulation, decreasing IL-1, IL-6, and TNFα levels [42]. The immunosuppressive outcome of HBOT might be owing to variations in the distribution of mononuclear cells and macrophage function impairment which is a significant origin of IL-1 and TNFα. Furthermore, HBOT was instituted to persuade apoptosis in other cytokine-producing cells reducing cytokine manufacture [43]. The immunosuppression of the pro-inflammatory interleukins IL-1 and IL-6 have beneficial effects in various inflammatory disorders including viral contagions [11, 12, 44]. HBOT declines TLR expression, NF-kB signaling trails, and the expression of these molecular platforms in diverse tissues [45, 46].
3. It has several bactericidal, bacteriostatic effects, suppresses toxin production, or strengthens resistance against contagions. HBOT inhibits the adherence of neutrophils to the endothelium of vessels, reducing inflammation, free radicals manufacture, vasoconstriction, and tissue destruction [47, 48].
4. It can noticeably prevent the variation in the coagulation cascade in an experimental model of multiple organ failure syndrome. In a study done by Imperatore et al. [49], they explained that HBOT could reduce the stimulation of the coagulation system, the inhibition of fibrinolysis, and platelet hyper-aggregation.
5. It has a great beneficial role in the management of various thrombotic events as cerebral thrombosis since it relieves brain edema by its vasoconstriction action and counteracts the vasodilatation of the capillaries in the hypoxic tissues decreasing the permeability of BBB. HBO also reduces the swelling of the neurons by improving their metabolism [50]. COVID-19 may persuade to both venous and arterial embolisms owing to excessive inflammation along with hypoxia, immobilization, and diffuse intravascular coagulation [51].

A recent systematic review summarized that overall, HBOT seems to be a safe and effective method of oxygenation in patients with COVID-19. However, its large space occupation and lack of availability in large numbers may limit its use in the settings of a pandemic where many patients require oxygenation, and this shortcoming needs to be addressed. There is limited knowledge and evidence regarding the effects of HBOT in the settings of COVID-19, and further well-designed trials with larger sample sizes are recommended to carefully assess the outcomes of this treatment modality and compare it with other oxygenation methods [52].

Management protocol of HBOT in COVID-19 according to the recent recommendations summarized that HBOT is indicated in confirmed COVID-19 patient with SO_2_ saturation ≤ 90%, with signs of hypoxemia or pulmonary hypoxia provided no respiratory distress, pulmonary shock, emphysema, air cysts, or bullae, and untreated pneumothorax. The treatment protocol is carried out with 1.45 atmospheric pressure sessions of 120 min, once per day. The patient and the operator must have all the equipment required for personal safety and isolation. Regular evaluation of oxygen saturation is essential; CT chest and ultrasound are obligatory to evaluate the clinical response to treatment after the 5th session [53].

There may also many side effects of HBOT such as middle ear barotrauma (MEB), sinus/paranasal barotrauma, dental barotrauma, central nervous system (CNS) oxygen toxicity, pulmonary oxygen toxicity, hyperoxic myopia, cataracts, retrolental fibroplasia following hyperoxic exposure, claustrophobia or increase in blood pressure, pulmonary edema, and hypoglycemia in diabetics [54]. Furthermore, oxygen toxicity due to excess free radical generation, barotrauma to the middle ear, pneumothorax, and inert gas uptake induced narcosis are the commonly reported complications of HBOT.

### N-acetyl cysteine (NAC)

**Fig. 1:**
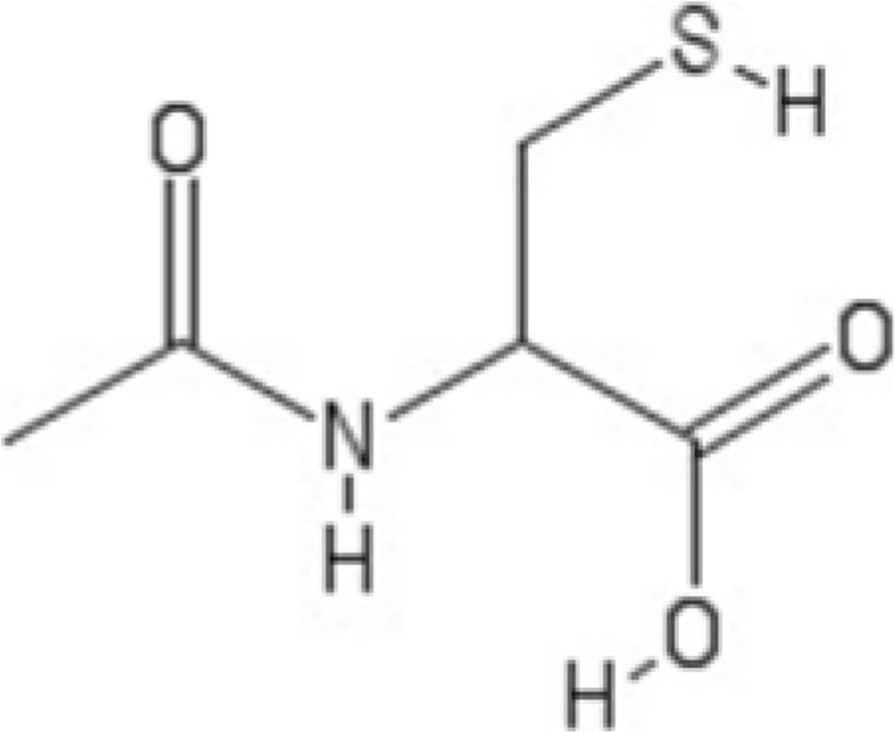
Structure of the N-acetyl cysteine (NAC)

It is an antidote of paracetamol overdose; it is an acetylated precursor of L-cysteine amino acid where acetyl group attached to the nitrogen atom. It has been also approved in the management of various disorders involving; doxorubicin cardiotoxicity, ischemia-reperfusion cardiac injury, bronchitis, acute respiratory distress syndrome (ARDS), HIV/AIDS, and psychiatric disorders [55] (Fig. 1).

The World Health Organization recognizes NAC as a pertinent and a significant medication required in a very basic health system. It may be administered orally or intravenously or in a nebulizer form as in cases of acute lung diseases [56]. It has numerous advantages: as is considered a stable drug, commercially available at a low price and a safe drug with low bioavailability [57].

### Mechanism of action

NAC has shown mucolytic, antioxidant, and anti-inflammatory properties.

1. Mucolytic effect

It has a mucolytic effect by breaking the disulfide bridges between macromolecules of mucous and reducing the heavily cross-linked mucus, resulting in condensed mucous viscosity [56, 58].

2. The antioxidant effect

N- Acetyl cysteine is a significant antioxidant and a cytoprotective mediator that reloads intracellular glutathione. It has been shown to have a defensive role against cardiovascular complications [55]. This action is through complex methods by acting as a glutathione precursor and being a precursor of a thiol group [59, 60].

The antioxidant effect can be related to at least three different mechanisms

I. *A direct antioxidant effect:* NAC can act as a direct antioxidant or direct scavenger for many oxidant radicals such as “NO_2_, ˙OH, and CO_3_” and non-radical oxidants such as hypochlorous (HOCl) and related species which are oxidants formed from activated neutrophils and monocytes through the activity of myeloperoxidase (MPO) that are involved in the pathophysiology of some lung diseases [61].
II. *An indirect antioxidant effect*: as a result of NAC's capability to act as a cysteine precursor, in which it serves as a substrate for the synthesis of glutathione (GSH) in the body, thus preventing the damaging effects in some organs like; liver, skin, lung, and brain [62].
III. GSH is a direct antioxidant for a lot of antioxidant enzymes including glutathione (reductase, peroxidase, glyoxalases 1 and 2, peroxiredoxin, transferase) and (membrane-associated proteins in eicosanoid and glutathione metabolism) MAPEG [63]*.* NAC also augments the glutathione content of the tissues and tissue GSH-to-oxidized GSH ratio [59, 60].
IV. A breaking effect on disulfides and reserve ability of thiol pools, which regulates the redox state; it is the unique reducing effect of NAC. This releases free thiols, which have better antioxidant activity and enhance the GSH synthesis, besides reduced proteins, which have an important direct antioxidant activity in numerous cases, as for mercaptoalbumin. The reducing action of NAC also can explain its mucolytic activity in reducing heavily cross-linked mucus glycoproteins [57, 64].

3. Anti-inflammatory effect

NAC inhibits the inflammatory cytokines including; tumor necrosis factor-alpha (TNFα), IL-1β, and IL-6. In addition, it can reduce the activation of transcription factors NF-κB which is responsible for the initiation of inflammatory process and causes downregulation of IL-10 mRNA and protein expression in NAC-treated cells which cause further modification of the inflammatory cytokine profile [56, 65].

#### Rationale of the potential efficacy of N acetyl cysteine in the management of COVID-19

NAC was described to reduce the manifestations of influenza-like illness in humans and administration of a dose of 100 mg/kg can contribute to the success of the management of contaminated cases with the 2009 pandemic H1N1 virus [66]. The following are the beneficial mechanisms of NAC in COVID-19

1. An anti-inflammatory effect is used in acute respiratory distress syndrome (ARDS) to protect cells from inflammation and apoptosis, so it may reduce the overall intensive care admission rate [67].
2. Antioxidant effect of NAC can potentially ameliorate COVID-19 induced oxidative stress complications as ARDS and multi-organ failure [68]. Also, the antioxidant effect of NAC can counteract the unfortunate antioxidant defense that present in patients with old age, smoking and chronic debilitating disorders, and may grave the outcome of COVID-19 due to the lack of endogenous glutathione [37, 38, 69]. proved that NAC increases the level and the activity of glutathione reductase (GR) enzyme, and an increased level of this enzyme can increase glutathione level in about 40% of COVID-19 cases.
3. Antiviral effects were supported by several studies that explaining its anti-viral activity against influenza A strains [70, 71]. It raises GSH levels which can diminish the viral load by inhibiting the viral replication, inhibits the production of pro-inflammatory molecules (CXCL8, CXCL10, CCL5 and interleukin-6 (IL-6)), and reduces the activation of transcription factors NF-κB [72].
4. NAC can ameliorate ribavirin therapeutic index, either by declining its toxicity or even allowing the use of a lower dose of the ribavirin, a previous report was conducted on mice illustrated that administration of NAC combined with antiviral therapy had a better response, both have a diverse mechanism of action that improves the outcome and reduce the severity of illness [71].

### NAC treatment regimen

NAC is recommended to use in high doses 600-900 mg twice daily in COVID -19 to decline the complications and to improve the outcome [68]. As NAC is considered a safe drug with low toxicity and mild side effects including nausea, vomiting, tachycardia, and pruritus, there is no fear to administrate it in high doses [55].

Lastly, Mohanty et al. [73] concluded that NAC has proven antioxidant activity which is useful in attenuating the immune activation and cytokine storm in animal and human studies. It is a safe, cost-effective, widely available drug, and its mechanism of action hypothetically suggests its potential role in the management of cytokine storm in COVID-19. However, with the limited evidence currently available, it would be imprudent to formulate any recommendation regarding the use of NAC in COVID-19. Any future recommendation regarding its use in this condition will depend on the outcome of the ongoing clinical trials. There are also found two case series related to the use of NAC in COVID-19 [74, 75].

### Naloxone and low-dose naltrexone (LDN)

#### Naloxone

Naloxone is an official primary antidote of opioid overdoes; it acts at the brain μ-opioid receptor as a neutral competitive antagonist [76], Naloxone has great selectivity for the μ opioid receptor ([77] ), in addition to its ability to bind to kappa and sigma opioid receptors. Besides its effects against exogenous opioids, the activity further extends to the endogenous endorphins [78]. It is one of the components of the “coma cocktail” which is a part of empirical treatments to correct altered mental status of unknown cause [79]. It can be used in the newborn for respiratory depression reversal that occurs due to trans-placentally acquired narcotics [80].

It was established that naloxone produces a favorable action on respiratory mechanics, pulmonary function test parameters, and oxygen metabolism of cases with respiratory failure, and it can considerably improve the general state of the body [81]. ChuiLi [82] reported that naloxone combined with the non-invasive ventilation can rapidly improve the clinical complaints and arterial blood gasses findings in respiratory failure associated with pulmonary encephalopathy*.* Newborns with respiratory failure also respond better with using naloxone in large doses, without any side-effects [83].

Ayres et al. [84] was the first one who explained the role of using naloxone in respiratory failure. Naloxone creates an increase in oxygen saturation in excess of the increase in ventilation, so better ventilation-perfusion matching is established in the acute illnesses with acute respiratory failure [84].

Chemically, Naloxone is a synthetic derivative of oxymorphone (C19H21NO4) in which the *N*-allyl group *replace N*-methyl one [42]. There are numerous routes in which naloxone can be administered, either intravenously, intramuscularly, subcutaneously, and intranasally. Oral forms of the drug has rapid first-pass metabolism and thus do not provide the necessary efficacy resulting in extensive naloxone metabolism before blood stream access [85]. The recommended original dose of naloxone ranges from 0.04 to 0.4 mg [86].

Naloxone had US Food and Drug Administration (FDA) approval in 1971 (off-patent 1985) as Narcan I.V. It is on the core list by WHO of the crucial medicines as it is regarded as an example of the ideal antidote [87]. Auto-injector naloxone has been approved by the US FDA for usage in 2014 (Edwards, [88]) as Evzio, and in November 2015, Narcan Nasal Spray became the first US FDA-approved non-injectable naloxone product for the treatment of opioid overdose [89].

Naloxone as a neutral antagonist, do not have risk for overdose [90] .In non-dependent opioids patients, naloxone has a high tolerability profile as opioid withdrawal is the most frequently reported adverse effect with symptoms of nausea, irritability, vomiting, and anxiety [91].

#### Low dose naltrexone (LDN)

Naltrexone has the highest μ-opioid receptors affinity, like naloxone being pure opioid antagonist that was approved by FDA for the treatment of opioid addiction in 1984 [92]. It is comparable to the naloxone concerning the structure and the function, but with better oral bioavailability and a longer biologic half-life [93]. Chemically, naltrexone is 17-(cyclopropylmethyl)-4,5-epoxy-3,14-dihydroxymorphinan-6-one [94].

The term low-dose naltrexone (LDN) refers to doses about 1/10th the size of the dose used normally in opioid overdose; typically 4.5 mg though a variable dose a limited milligrams below or above that communal value (50 mg) [95, 96]. In the contrary to higher doses of naltrexone, LDN cause increasing the endorphins release in the body through its action on the β-endorphin receptors [97].

##### Rationale of the potential efficacy of LDN in the management of COVID-19

1. LDN has the ability to reduce pro-inflammatory cytokines as it was found to be allied with a decline of plasma concentrations of transforming growth factor (TGF)-β which belongs to a group of cytokines that is together called ‘The super-family TGF-β’, and also responsible for regulating epithelial cell differentiation, expansion , organization, motility, and apoptosis [98].
2. LDN has the capability to raise anti-inflammatory cytokines. It is established that COVID-19 cases were allied with increased level and activity of TGF-β due to the virus-induced violent immune and inflammatory reactions with the dysregulation of the coagulation and fibrinolytic pathways that extremely stimulate the latent TGF-β in the lungs and the blood of the contaminated cases [99]. COVID-19 contagion is linked with massive increase of neutrophil infiltration into the lungs where the neutrophils can release the stored TGF-β, being a strong chemokine-like molecule; so, it can engage more neutrophils into the lung resulting in a vicious cycle. LDN can ameliorate edema and fibrosis in the lungs of COVID-19 cases, due to activation of TGF-β which is one of the known causes of lung fibrosis and as well as disturbances in the fluid homeostasis in the lung [100]; due to uncontrolled inflammatory reactions, “cytokine storm” ultimating in edema and fibrosis in the lungs in COVID-19 cases [101].
3. LDN is an immune-modulator that decreases the number of TH1 cells and TH17 cells results in immune–balance with the regulation of cytokines release [36]. Among cytokines concerned in the storm allied with COVID-19 are those involved in T helper 17 (TH17) type responses as evidenced by the remarkably high number of TH17 cells in the peripheral blood of a case with severe COVID-19 contagion [35]. TH17 cells themselves produce IL-17, G-CS, IL-21, IL-22, IL-17 IL-1β, IL-6, and TNFα interleukins. IL-17 has extensive pro-inflammatory properties on the induction of cytokine G-CS (responsible for recruitment of neutrophils) and IL-22 (which stimulates mucins, fibrinogen, anti-apoptotic proteins, and serum amyloid A) [102].
4. LDN is a modulating tool of the neuroimmune axis causing higher reactivity of immune cells mediated by transient increases in the opioid growth factor [103, 104] . The classical effect of naltrexone at low-dose range is the transient opioid receptor blockade resulting in up-regulation of opioid signaling [105]. Experimental models guarantee the role of LDN in the upregulation of the endogenous opioid system through raising the levels of beta-endorphin and met-enkephalin (opioid growth factor) [106].
5. LDN is considered as “Enhancer of quality of life” due to its probable neuropsychological theoretical benefits [105] as upregulated endorphins have neuropsychological benefits arises from the well-reported relations between mu-opioid receptors and mesencephalon dopamine neurons [103, 107]. The field of psychoneuroimmunology (PNI) illustrates the link between mood and immunity allowing mutual influences between the brain and the immune system [108, 109].
6. LDN may promote both the prevention and the management of viral disorders and bacterial contagions through being immune functions enhancer and especially the natural killer cell activity [110]. Cytokines as chemical messengers are made by immune cells that can be either increase or decrease the immune function. The body’s capability to keep a balance between cytokines that promote inflammation and those that reduce it is responsible for the harmonization between the diverse responses of the immune system [111].

#### The potential side effects of naloxone in the context of COVID-19

A recent meta-analysis focused on probable troubles subsequent to naloxone management, precisely reviewing literature related to whether naloxone increased the risk of seizures after treatment of tramadol poisoning [112]. Furthermore, a recent study in 2017 stated on 2 unblinded randomized controlled articles comparing the frequency of adverse measures with either intranasal naloxone via a mucosal atomizer or intramuscular naloxone, involving anxiety and/or violence, nausea, vomiting, and headache [113].

#### Deferoxamine (Desferal)

Deferoxamine is an iron-chelating mediator used in the handling of acute iron poisoning and chronic iron overload. It belongs to heavy metal antagonists as it chalets iron by creating a stable complex which averts additional biochemical reactions of iron. It is available in vials for any form of injection.

##### Rationale of the potential efficiency of deferoxamine in the management of COVID-19

1. Decreasing the iron availability by deferoxamine causing decline of viral multiplication [114]. Furthermore, it is declared that iron overload is also implicated as a hazard element for rapid evolution of the illness [115].
2. Inhibiting the synthesis of DNA through inactivation of iron-dependent ribonucleotide reductase, thus altering the viral multiplication [116].
3. Reducing the development of free hydroxyl radicals [117]. Deferoxamine is a well-known antioxidant agent. High serum level of iron is allied with increased oxidative stress particularly in viral-infected cases [118, 119].
4. Ameliorating the cardiac injuries of severely ill COVID-19 cases throughout its antioxidant features [14].
5. Preventing the organ injury and reduce the fatality rate in a variety of experimental and clinical models of ischemia–reperfusion injury [120].

#### Adverse effects

- Local responses such as swelling and tenderness at the injection site
- Systemic reactions for example allergic reactions, arthralgia, fever, headache, myalgia, or asthma
- Digestive troubles as abdominal ache, nausea, vomiting, and diarrhea
- Cardiovascular reactions as tachycardia, shock, and hypotension
- Other adverse properties like blood dyspraxia, cramps in the leg, and some neurological disorders involving faintness, peripheral neuropathy, paresthesia, and encephalopathy
- Ocular and auditory dilemma

**Fig. 2:**
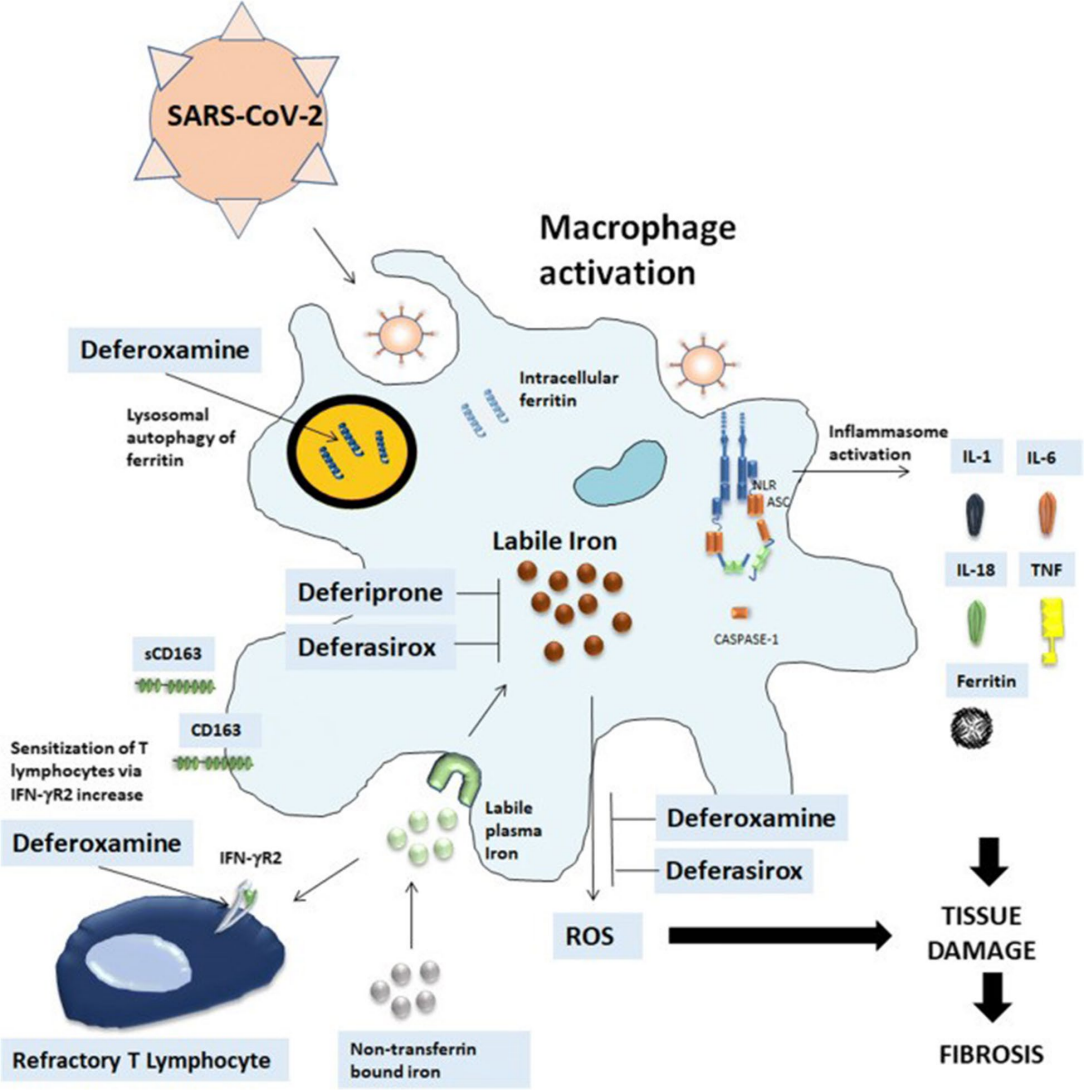
Deferoxamine action (https://europepmc.org/article/med/32681497#)

Desferal-iron complex is excreted predominantly throughout the kidney, so it is contraindicated in cases with severe renal troubles (Fig. 2).

## Conclusions

- Deferoxamine is an iron-chelating agent. The use of deferoxamine seems to be of very limited benefit in selected patients in critical COVID-19 with vague mechanism.it needs to be validated especially that its adverse effects are multiple and may be fatal.
- Besides the antidotal action of naloxone and naltrexone as a pure opioid antagonist, naloxone may show some benefit but still there is limited evidence-based information about this and the authors didnot mention the potential side effects of the medication in the context of COVID-19. Maybe its use in critical or severe COVID may be beneficial but still this needs validation.
- Hyperbaric oxygen therapy (HBOT) is the most powerful oxygen therapy known. It can solve the problem of hypoxemia more effectively than normal pressure oxygen therapy with either high flow oxygen inhalation or mechanical ventilation as well as its role in improving circulation, alteration of coagulation cascade, and immune suppressive effect. Moreover, it seems that this is a rather expensive modality of treatment for very selected patients that is not available in most centers and requires special expertise. Obviously, the pandemic was very widespread rendering the use of this modality tremendously limited.
- N. acetyl cysteine is considered an essential drug for a healthy life, a safe drug with mild side effects. There is so limited evidence-based proof for the benefit of N-acetyl cysteine in treatment of mild and moderate cases of COVID-19 and although side effects are not serious but they really upset the patient especially the GIT symptoms.
- Finally, further large clinical trials are needed to study the efficacy of these agents.

## Data Availability

Data are available.

## Abbreviations

SARS-CoV: Severe acute respiratory syndrome coronavirus
MERS-CoV: Middle East respiratory syndrome coronavirus
COVID-19: Coronavirus disease 2019
WHO: World Health Organization
nCoV-19: Novel coronavirus
HCoV-229E: Human coronavirus 229E
HCoV-OC43: Human coronavirus OC43
SARS: Severe acute respiratory syndrome
ICU: Intensive care unit
HBOT: Hyperbaric oxygen therapy
TNFα: Tumor necrosis factor alpha
TLR: Toll-like receptor
NF-κB: Nuclear factor κB
PE: Pulmonary embolism
IHBO: Intermittent hyperbaric oxygen
HBO: Hyperbaric oxygen
BBB: Blood-brain barrier
SO2: Oxygen saturation
NAC: N-acetylcysteine
NO2: Nitrogen dioxide
OH: Hydroxyl radical
CO3˙̄: Carbonate radical anion
HOCl: Hypochlorous acid
GSH: Glutathione
IL10: Interleukin 10
ARDS: Acute respiratory distress syndrome
CXCL8: Chemokine (C-X-C motif) ligand 8
CXCL10: C-X-C motif chemokine 10
CCL5: Chemokine (C-C motif) ligand 5
LDN: Low-dose naltrexone
US: United States
FDA: Food and Drug Administration
TGF: Transforming growth factor
TH: T helper cell
G-CS: Granulocyte colony stimulating
